# Highlighting the Importance of Authentic Reference Chemicals in the Unambiguous Identification of Unknowns during Routine Sample Analysis—An OPCW Proficiency Test Case

**DOI:** 10.1021/jasms.6c00191

**Published:** 2026-07-13

**Authors:** Carolyn J. Koester, Teneile M. Alfaro, David S. Cho, Todd H. Corzett, Mark L. Dreyer, Saphon Hok, Derrick C. Kaseman, Adele F. Panasci-Nott, Alexander K. Vu, Armando Alcaraz, Carlos A. Valdez

**Affiliations:** † Physical and Life Sciences Directorate, 4578 Lawrence Livermore National Laboratory, Livermore, California 94550, United States; ‡ Biosciences and Biotechnology Division, Lawrence Livermore National Laboratory, Livermore, California 94550, United States; § Forensic Science Center, Lawrence Livermore National Laboratory, Livermore, California 94550, United States; ∥ Nuclear and Chemical Sciences Division, Lawrence Livermore National Laboratory, Livermore, California 94550, United States; ⊥ Materials Science Division, Lawrence Livermore National Laboratory, Livermore, California 94550, United States

## Abstract

The unequivocal identification of unknown chemicals during routine sample analysis is a constant occurrence in the field of analytical chemistry. The process can be painstakingly tedious, particularly in situations where a tentatively identified unknown may possess isomeric counterparts yielding identical accurate mass values and very similar mass spectra. In this work, we present the experimental process involved in the correct identification of 1,4-oxathiane 4,4-dioxide and differentiation from its constitutional isomer, 2-hydroxyethyl vinyl sulfone, when present in a silica gel matrix featured in the 54th Environmental Organisation for the Prohibition of Chemical Weapons (OPCW) proficiency test. After discovering that the library-generated mass spectrum for 2-hydroxyethyl vinyl sulfone in a preliminary gas chromatography–mass spectrometry (GC-MS) analysis did not match the one from an authentic reference chemical, we embarked on an unknown structure determination campaign involving GC-MS, liquid chromatography-tandem mass spectrometry (LC-MS-MS), and nuclear magnetic resonance (NMR) spectroscopy. All of the information obtained, coupled to the synthesis of an authentic reference chemical, was used to determine the identity of the unknown as 1,4-oxathiane 4,4-dioxide. The process described in this work highlights the important role played by authentic reference chemicals in the identification of unknown chemicals during routine sample analysis.

## Graphical abstract

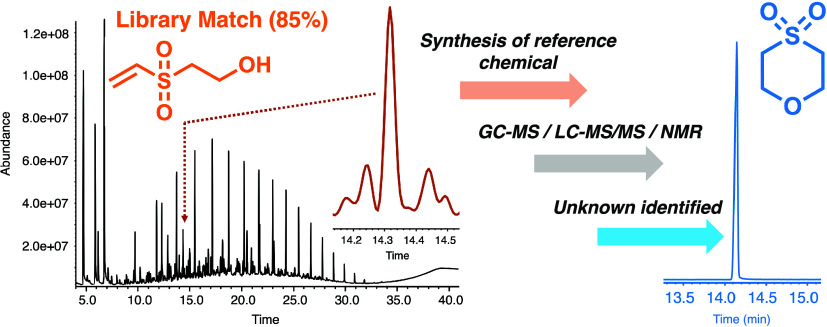

## Introduction

In 1997, the Chemical Weapons Convention (CWC) started the worldwide, concerted effort for the global eradication of chemical warfare agents (CWAs).^1^ As a practical part of this operation, the CWC also set in motion the careful monitoring and control of chemicals that can be used as precursors to produce CWAs, a task that the Organisation for the Prohibition of Chemical Weapons (OPCW) is responsible for enforcing.^2,3^ These chemicals were organized into scheduled groups depending on their association with CWAs, ranging from Schedule 1 to Schedule 3. Chemicals in Schedule 1 include the actual CWAs, while those in Schedule 2 comprise other toxic chemicals and Schedule 1 precursor chemicals. Purchase or acquisition of Schedule 2 chemicals has become tightly regulated by agencies worldwide. Lastly, Schedule 3 chemicals can also be used as chemical agent precursors; however, they are also produced commercially in large quantities for legitimate industrial uses.^3^ For this reason, they are less tightly controlled than the other scheduled chemicals. Detection of any scheduled chemicals or their degradation products during an inspection by a team of forensic scientists working on behalf of the CWC might suggest the production or use of CWAs at a given location.

The detection of chemicals relevant to the CWC presents a challenge. First, the CWC does not include a complete list of relevant chemicals, but, rather, lists chemical classes. For example, the nerve agent Sarin is only one compound belonging to Schedule 1.A.01, which is described by the CWC as “O-Alkyl (≤C10, incl. cycloalkyl) alkyl (Me, Et, *n*-Pr or *i*-Pr)-phosphonofluoridates”; thus, hundreds of chemicals comprise this class. Second, the CWC continues to grow to accommodate newly emerging CWAs as well as chemicals involved in their production.^3^ Third, chemicals of relevance to the CWC have a wide range of physical properties, meaning that no single analytical technique can be used to detect all of them. Due to the ability to create reproducible libraries and its maturity as an analytical technique, gas chromatography–mass spectrometry with electron ionization [GC-MS (EI) or GC-MS] is usually the first tool applied to the detection of Scheduled chemicals in a collected environmental sample.^4^ However, polar chemicals, arising from the degradation of CWAs or which constitute building blocks for their production, often require derivatization before detection by GC-MS.^4–8^ For these polar chemicals, liquid chromatography–mass spectrometry (LC-MS) is often used for their direct detection. However, LC-MS has the limitation that not all polar chemicals ionize efficiently in order to be detected by atmospheric pressure ionization techniques. For example, pinacolyl alcohol (a precursor of the nerve agent Soman)^5^ and cyclic sulfone and sulfoxides (indicators of the blister agent sulfur mustard) are not amenable to analysis by LC-MS. Thus, laboratories working to analyze chemicals of relevance to the CWC must have access to a diverse set of analytical tools, including derivatization strategies, to use in conducting their work.

## The Purpose and Structure of an OPCW Proficiency Test

OPCW’s network of designated laboratories is an essential element of the Organisation’s verification regime and its capacity to investigate allegations of the use of CWAs. To maintain good standing as a designated laboratory of the OPCW, member laboratories around the world are subject to yearly proficiency tests (PTs). In each PT, several reportable chemicals as chosen by the sample preparation laboratory are spiked into a solid and/or a liquid matrix at concentrations that vary from 1 to 50 ppm.^9^ In addition to these reportable chemicals, interferences (e.g., diesel fuel or polyethylene glycols) are often added to make the identification of a reportable compound extremely difficult. During these PTs, the detection of a reportable chemical, and its identification and confirmation by using two or more analytical techniques, is imperative for successfully completing the test. If a reported chemical is incorrectly identified, the laboratory will fail the PT and, thus, lose its designation status.

Most designated laboratories use a similar strategy to approach PT sample analysis.^4^ Samples are prepared by multiple methods and analyzed with different analytical techniques. Typically, GC-MS (EI) is initially used to screen for reportable compounds—exploiting the use of mass spectral databases and library matching (e.g., NIST and AMDIS) to rapidly obtain a preliminary identification of a reportable chemical.^10,11^ In most cases, the spectral matching is successful at guiding the analysts’ subsequent analytical work using other methods such as LC-MS or other hyphenated GC-based methods (e.g., GC coupled with element-specific detection, chemical ionization mass spectrometry, or high-resolution mass spectrometry). The use of a mass spectral library provides a great advantage in most cases, as even if a matching score is not convincing enough to identify a chemical, it provides the analytical team with a limited number of potential compounds to synthesize for comparison (i.e., reference chemicals). To unambiguously identify a chemical, an authentic reference chemical must be analyzed under the same conditions as the sample to demonstrate that its retention time and mass spectrum match those of the unknown chemical being identified in a sample. Given the potentially serious consequences of reporting chemicals relevant to the CWC, it is imperative that there are no mistakes in their identifications.

The purpose of the case presented in this paper is to highlight the critical role authentic reference chemicals play in the unambiguous identification of an unknown chemical during sample analysis. In this work, we describe a scenario encountered as our laboratory participated in a recent PT. Our aim is to describe the process from the preliminary identification of an unknown chemical using mass spectral matching to the use of orthogonal methods to confirm its structure to its unequivocal identification by comparison to an in-house synthesized reference chemical. This example serves as a cautionary tale that solely relying on a library match from databases for chemical identification is not sufficient and that multiple analytical techniques are essential in providing unambiguous chemical identifications.

## A Challenge Arises during an OPCW PT

The 54th Environmental OPCW PT reminded us of the importance of synthesizing or procuring authentic reference chemicals for confidently confirming the identity of unknown chemicals spiked in various matrices.^12,13^ During this PT, six samples were received for analysis: three simulated solid waste samples (silica gel matrix) and three liquids. Initial analysis of an extraction of the solid sample (designated “S1”) by GC-MS revealed the existence of a compound (retention time: 14.3 min) tentatively identified by the NIST library match as 2-hydroxyethyl vinyl sulfone (CAS #: 4271–48–1) (Figure 1a,b). The match value for this unknown compound to the NIST library was high (R. Match: 947; 85% probability). As part of our routine protocols during a PT, our group undertook the synthesis of 2-hydroxyethyl vinyl sulfone^14^ (Supporting Information, Scheme S1, Page S6) for comparison to the unknown chemical found in sample S1. 2-Hydroxyethyl vinyl sulfone was synthesized by the base-promoted hydrolysis of 2-chloroethyl vinyl sulfone (CAS #: 7327–58–4). The most optimal conditions for the synthesis of 2-hydroxyethyl vinyl sulfone involved the treatment of 2-chloroethyl vinyl sulfone with 0.1 N NaOH at room temperature in acetonitrile (41% yield) (Supporting Information, Table S1, page S6).^15^ However, GC-MS analysis of the synthesized chemical yielded a different retention time and a different mass spectrum from the one exhibited by the unknown compound; in fact, the best NIST library match for the synthesized reference chemical 2-hydroxyethyl vinyl sulfone was the one for bis­(2-hydroxyethyl) sulfone (CAS #: 2580–77–0) (R. Match: 697; 64.8% probability) (Figure 1d).

**Figure 1:**
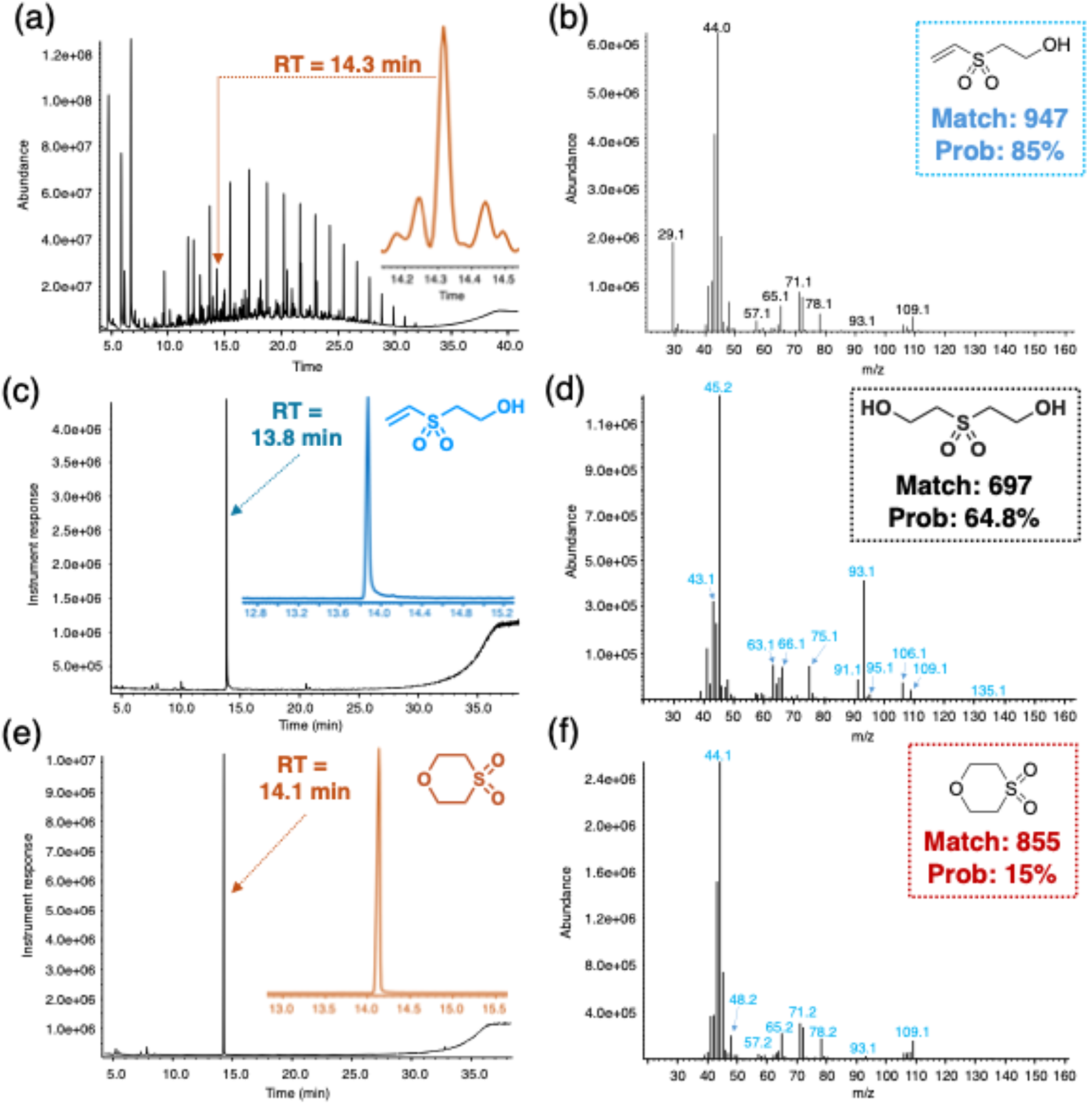
(a) GC-MS (EI) total ion chromatogram (TIC) of S1 sample with the unknown chemical indicated by the arrow and expanded in the inset, (b) mass spectrum for unknown chemical and NIST library matching parameters in inset yielding 2-hydroxyethyl vinyl sulfone as the probable candidate responsible for the spectrum, (c) GC-MS (EI) TIC of 2-hydroxyethyl vinyl sulfone reference chemical, (d) mass spectrum of 2-hydroxyethyl vinyl sulfone reference chemical, (e) GC-MS (EI) TIC of 1,4-oxathiane 4,4-dioxide reference chemical, and (f) mass spectrum of 1,4-oxathiane 4,4-dioxide reference chemical.

## Reference Chemicals Provide Crucial Information during Unknown Analysis

The aforementioned observations immediately prompted the reevaluation of the data obtained from the original GC-MS analysis and additional analyses of sample S1 carried out by other analytical methods. Further analysis using gas chromatography–mass spectrometry with chemical ionization [GC-MS (CI)] and gas chromatography-tandem mass spectrometry with chemical ionization [GC-MS-MS (CI)] revealed that the molecular ion of the unknown chemical in sample S1 was [M + H]^+^ = 137, suggesting a molecular weight of 136 (Supporting Information, page S2). The results of LC-MS-MS analysis using a high-resolution Orbitrap mass spectrometer (which also allowed us to collect LC-MS accurate mass data for the unknown chemical) supported the GC-MS-MS (CI) data, showing that the [M + H]^+^ ion was 137.0267 with a chemical formula of C_4_H_9_SO_3_ (calculated value for [M + H]^+^ ion of 137.0268, Supporting Information, Figure S6). These data sets opened the possibility of an isomeric compound related to 2-hydroxyethyl vinyl sulfone, yielding 1,4-oxathiane 4,4-dioxide (CAS no. 107–61–9) as a potential candidate. 1,4-Oxathiane 4,4-dioxide is a constitutional isomer of 2-hydroxyethyl vinyl sulfone, and it is its cyclic nature that provides the double-bond equivalency that allows for both compounds to have the same molecular formula. Although 1,4-oxathiane 4,4-dioxide is nonscheduled, it is a reportable compound by OPCW guidelines because it is a degradation product of the blister agent bis­(2-chloroethyl)­sulfide.^16^ This highlighted the urgent need to confirm its existence in the S1 sample. 1,4-Oxathiane 4,4-dioxide was synthesized via oxidation of 1,4-oxathiane using various oxidants, with 30% H_2_O_2_ being the most optimal one at producing a mixture of the sulfoxide as well as the fully oxidized sulfone (Supporting Information, Table S2, Page S7). GC-MS analysis of the 1,4-oxathiane 4,4-dioxide after purification confirmed that this chemical possessed a matching mass spectrum to the S1 unknown compound (Figure 1e,f and Supporting Information, Figures S3 and S4). However, as observed in Figure 1f, the NIST library match for this chemical was not as expected, attributing its mass spectrum to that of 2-hydroxyethyl vinyl sulfone (match: 855; 15% probability). Additional comparative analyses of mass fragments for both reference chemicals show several spectral differences, and these are presented in the Supporting Information (page S7).

## Further Confirmation of Unknown’s Structure Is Established

Orthogonal supporting data obtained using LC-MS-MS also provided direct evidence that the unknown compound was indeed 1,4-oxathiane 4,4-dioxide, as its retention time during the analysis matched the retention time of the authentic reference chemical as well as its MS-MS spectra (Supporting Information, Figures S5–S8, Pages S4–S5). Further evidence that the unknown analyte in sample S1 was 1,4-oxathiane 4,4-dioxide and not 2-hydroxyethyl vinyl sulfone was provided by a proton nuclear magnetic resonance (^1^H NMR) spectroscopy experiment on sample S1 (H_2_O suppression) wherein the vinyl protons in 2-hydroxyethyl vinyl sulfone were not found within the expected chemical shift range (i.e., between 5.0 and 7.0 ppm) (Supporting Information, Figure S9, Page S8). Nevertheless, the methylene proton signals arising from the reference chemical cyclic 1,4-oxathiane 4,4-dioxide were found to match those found in sample S1 (i.e., resonances at δ 3.13 and 4.15 ppm) (Supporting Information, Figure S10, Page S8). One last important observation during the early-stage analysis of sample S1 was that the unknown compound did not undergo derivatization with *N,O*-*bis*(trimethylsilyl)­trifluoroacetamide (BSTFA).^17^ This observation strengthened our doubts about the accuracy of the original NIST library match, as the hydroxyl group in 2-hydroxyethyl vinyl sulfone would be expected to undergo silylation, and thus, the unknown compound would have experienced a noticeable shift in its retention time and also a change in its mass spectrum. With the current knowledge that the unknown chemical was 1,4-oxathiane 4,4-dioxide, this initial observation makes perfect sense, as this chemical is not going to undergo a derivatization reaction with BSTFA. Therefore, based on the summation of collected data, our team determined that the unknown reportable compound was 1,4-oxathiane 4,4-dioxide and was included as such in the final report. Evaluation of our PT results found that this identification was correct, and our laboratory retained its good standing within the OPCW designated laboratory community.

## Concluding Remarks

The process used to unambiguously determine the identity of an unknown chemical during an OPCW PT illustrates that it is not sufficient to identify a compound based solely on a library match to a database using a single analytical technique (and, in this case, we also discovered that one of the mass spectral database entries was incorrect). Further work on reliably deducing the identity of an unknown chemical requires the performing laboratory to combine data from multiple analytical techniques such as LC-MS-MS and NMR and to ensure a good comparison of all data to that of an authentic reference chemical for an unambiguous confirmation. The situation described herein serves as a reminder of the central role of synthetic reference chemicals in the chemical identification. These reference chemicals, usually made on-demand due to pressing timelines or commercially available, provide analysts with a high degree of confidence when identifying an unknown chemical.

## Supplementary Material



## Data Availability

The data that support the findings of this study are available within the manuscript as well as in the Supporting Information section.

## Notes

EI-GC-MS and GC-MS-MS analyses on S1 matrix; LC-MS-MS analyses on S1 matrix; chemical synthesis of 2-hydroxyethyl vinyl sulfone and 1,4-oxathiane 4,4-dioxide standards; and nuclear magnetic resonance data on standards and S1 matrix (PDF)

## Abbreviations

AMDIS: automated mass spectral deconvolution and identification system
CWA: chemical weapons agent
CWC: chemical weapons convention
GC-MS (CI): gas chromatography–mass spectrometry with chemical conization
GC-MS (EI): gas chromatography–mass spectrometry with electron ionization
GC-MS-MS (CI): gas chromatography-tandem mass spectrometry with chemical ionization
LC-MS: liquid chromatography mass spectrometry
LC-MS-MS: liquid chromatography-tandem mass spectrometry
NIST: National Institute of Standards and Technology
NMR: nuclear magnetic resonance (spectroscopy)
OPCW: Organisation for the Prohibition of Chemical Weapons
PT: proficiency test
