# Supplementary material for: Highlighting the Importance of Authentic Reference Chemicals in the Unambiguous Identification of Unknowns during Routine Sample Analysis—An OPCW Proficiency Test Case
Source: J Am Soc Mass Spectrom. 2026 Jul 13;37(8):1718–23. doi: 10.1021/jasms.6c00191 (PMC13450385; doi:10.1021/jasms.6c00191)
Supplement: Supplementary file 1 [file js6c00191_si_001.pdf]

-Supporting Information-

"Highlighting the importance of authentic reference chemicals in the unambiguous identification of unknowns during routine sample analysis - An OPCW proficiency test case"

Carolyn J. Koester[1,3,4]*, Teneile M. Alfaro[1-3], David S. Cho[1,3,4], Todd H. Corzett[1-3], Mark L. Dreyer[1,3,5], Saphon Hok[1,3,4], Derrick C. Kaseman[1,3-5], Adele F. Panasci-Nott[1,3,5], Alexander K. Vu[1,3-5], Armando Alcaraz[1,3,4,]*, Carlos A. Valdez [1-3,]*

[1]Physical and Life Sciences Directorate, Lawrence Livermore National Laboratory, Livermore, CA, 94550, USA.
[2]Biosciences and Biotechnology Division, Lawrence Livermore National Laboratory, Livermore, CA, 94550, USA.
[3]Forensic Science Center, Lawrence Livermore National Laboratory, Livermore, CA, 94550, USA.
[4]Nuclear and Chemical Sciences Division, Lawrence Livermore National Laboratory, Livermore, CA, 94550, USA.
[5]Materials Science Division, Lawrence Livermore National Laboratory, Livermore, CA, 94550, USA.

*To whom correspondence should be addressed:
Dr. Carolyn J. Koester (koester1@llnl.gov); Mr. Armando Alcaraz (alcaraz1@llnl.gov); Dr. Carlos A. Valdez (valdez11@llnl.gov).

Table of Contents

	Page
Experimental Methods Description	S2
Chemicals and reagents	S2
EI-GC-MS Analysis	S2
GC-MS-MS Analysis	S2
Figure S1	S3
Figure S2	S3
Figure S3	S4
Figure S4	S4
LC-MS Analysis	S5
Figure S5	S5
Figure S6	S5
Figure S7	S6
Figure S8	S6
Synthesis of 2-hydroxyethyl vinyl sulfone	S7
Synthesis of 1,4-oxathiane 4,4-dioxide	S7
Comparative analysis of mass spectra	S8
Nuclear Magnetic Resonance	S9
Figure S9	S9
Figure S10	S9

Experimental Methods

Chemicals and reagents. All chemicals were purchased from commercial suppliers and used as received. Acetonitrile, dichloromethane, methanol, 2-chloroethyl vinyl sulfone, 1,4-oxathiane 4,4-dioxide, 1,4-oxathiane, *m*-chloroperbenzoic acid (mCPBA) and 30% hydrogen peroxide solution were purchased from Sigma-Aldrich (St. Louis, MO.). Sodium bicarbonate, sodium hydroxide, dimethylformamide, chloroform and anhydrous sodium sulfate were purchased from Acros Organics (Westchester, PA.). Autosampler vials and glass inserts were purchased from Agilent Technologies (Santa Clara, CA.). Thin layer chromatography (TLC) was used to monitor the extent of the oxidations using Merck Kieselgel 60-F_{254} sheets and detection accomplished with UV light (λ = 254 nm) in conjunction with development of color with ceric ammonium molybdate and iodine vapor.

GC-MS (EI) Analysis Method. A 6890 Agilent GC with a 5975 MS detector equipped with a split/splitless injector was used for all analyses as previously described. The GC column used for the analyses was an Agilent HP-5ms UI capillary column (30 m × 0.25 mm id × 0.25 µm film thickness). Ultra-high purity helium, at a 0.8 mL/min flow, served as the carrier gas. The inlet was operated in pulsed splitless mode (25 psi for 1 minute, followed by a 50 mL/min purge flow). The injector temperature was set at 250 °C and the injection volume used was 1 µL. The oven temperature program was as follows: 40 °C, held for 3 minutes, increased at 8 °C/min to 300 °C and held at this temperature for 3 minutes. The MS ion source and quadrupole temperatures were 230 °C and 150 °C, respectively. Electron impact (EI) was used with an ionization energy of 70 eV. The MS was operated to scan from m/z = 29 to 600 in 0.4 sec with a solvent delay of 3.5 minutes.

GC-MS-MS Chemical Ionization (CI) Analysis

GC-MS-MS (CI) analysis was performed on a ThermoScientific Trace 1310 instrument with a ThermoScientific TSQ 9000 detector equipped with a split/splitless injector was used for the analysis. The GC column used for the analysis was an Agilent HP-5MS UI: (5%-phenyl)-methylpolysiloxane column (30 m x 0.25 mm id x 0.25 µm i.f.). Ultra-high purity helium was used as the carrier gas at a 1.0 mL/min with a solvent delay of 3 min. The injection mode was splitless (0.75 min). The injector temperature was 250°C and the injection volume was 1 µL. The oven temperature program was as follows: 40°C, held for 3 min, increased at 8°C/min to 320°C, held for 3 min. The MS ion source temperature was set at 150°C. Electron ionization energy of 70 eV was used, and the polarity was positive. The reaction gas used was methane at a flow of 1.75 mL/min. The MS was operated to scan from *m/z* = 50 to *m/z* = 200 in 0.2 sec. The collision gas was argon, with a collision energy of 20 eV and a precursor ion of m/z = 137.0.

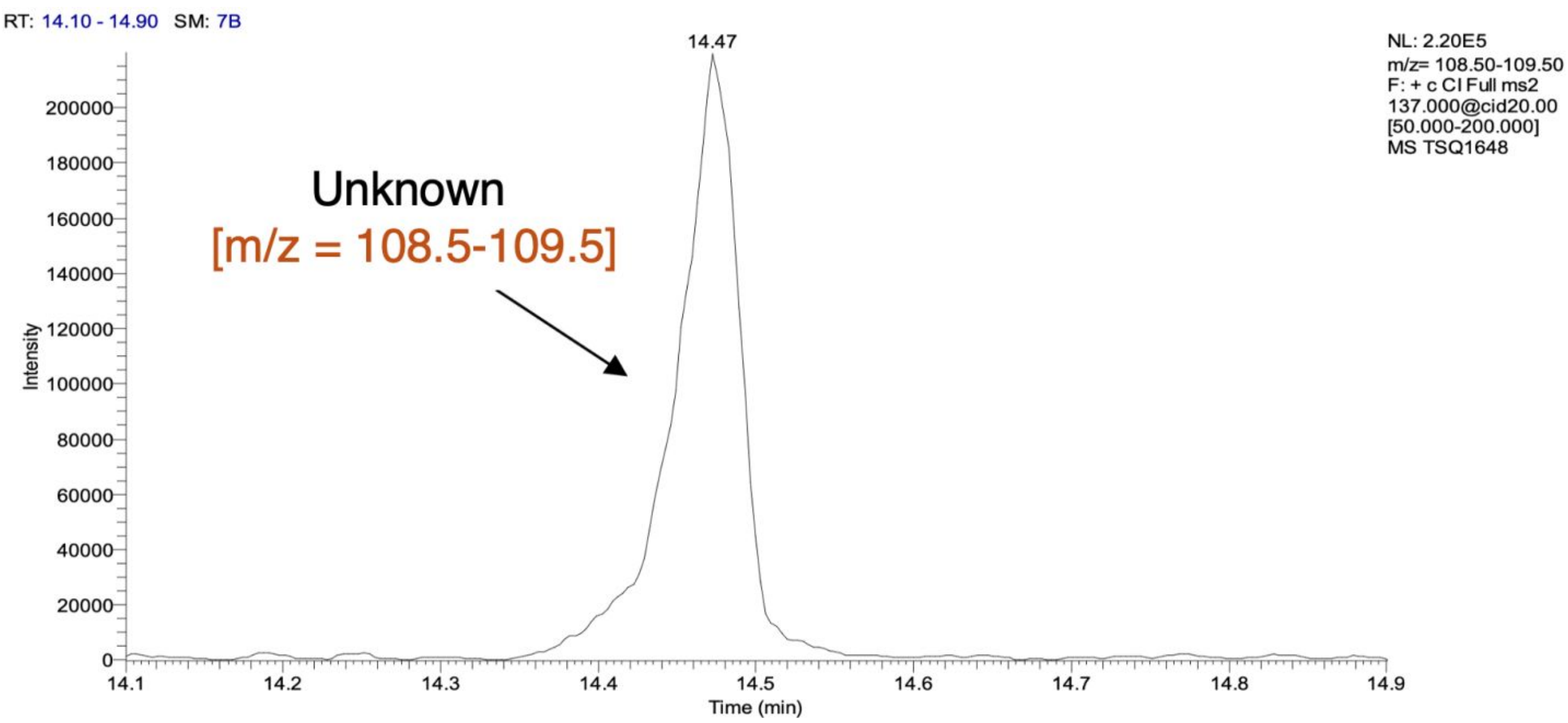

Figure S1. GC-MS-MS (CI) selected ion chromatogram for the unknown compound in the sample S1.

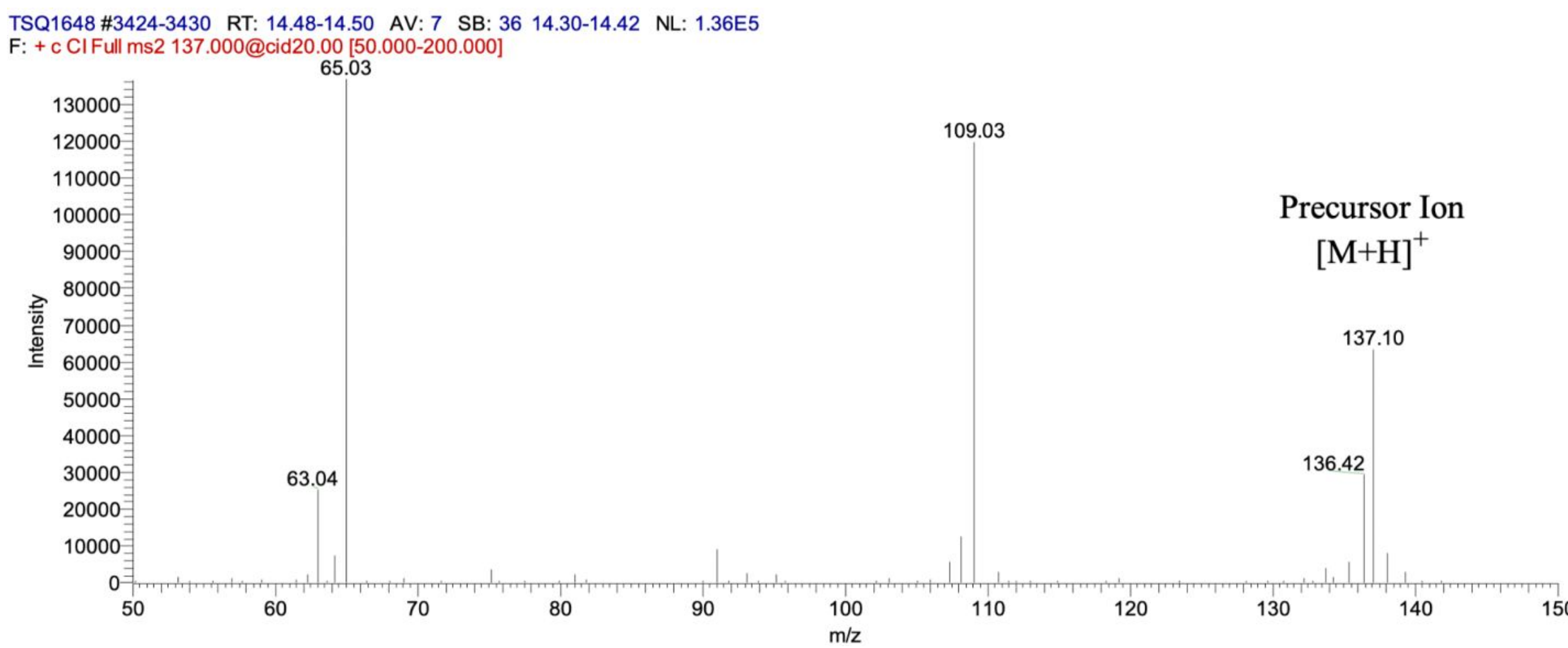

Figure S2. GC-MS-MS (CI) mass spectrum of unknown compound showing the $[M+H]^+$ precursor ion.

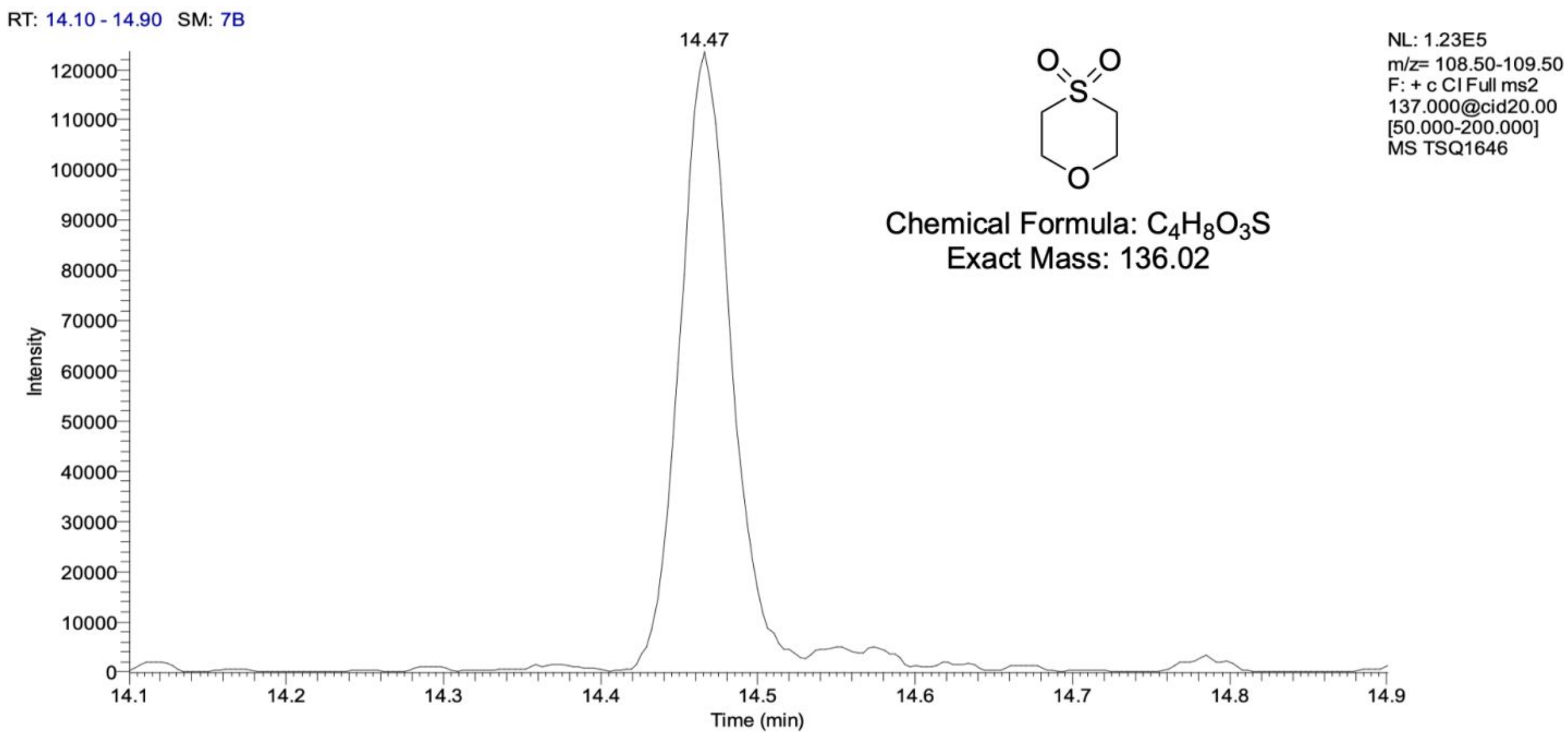

Figure S3. GC-MS-MS (CI) selected ion chromatogram for the 1,4-oxathiane 4,4-dioxide standard.

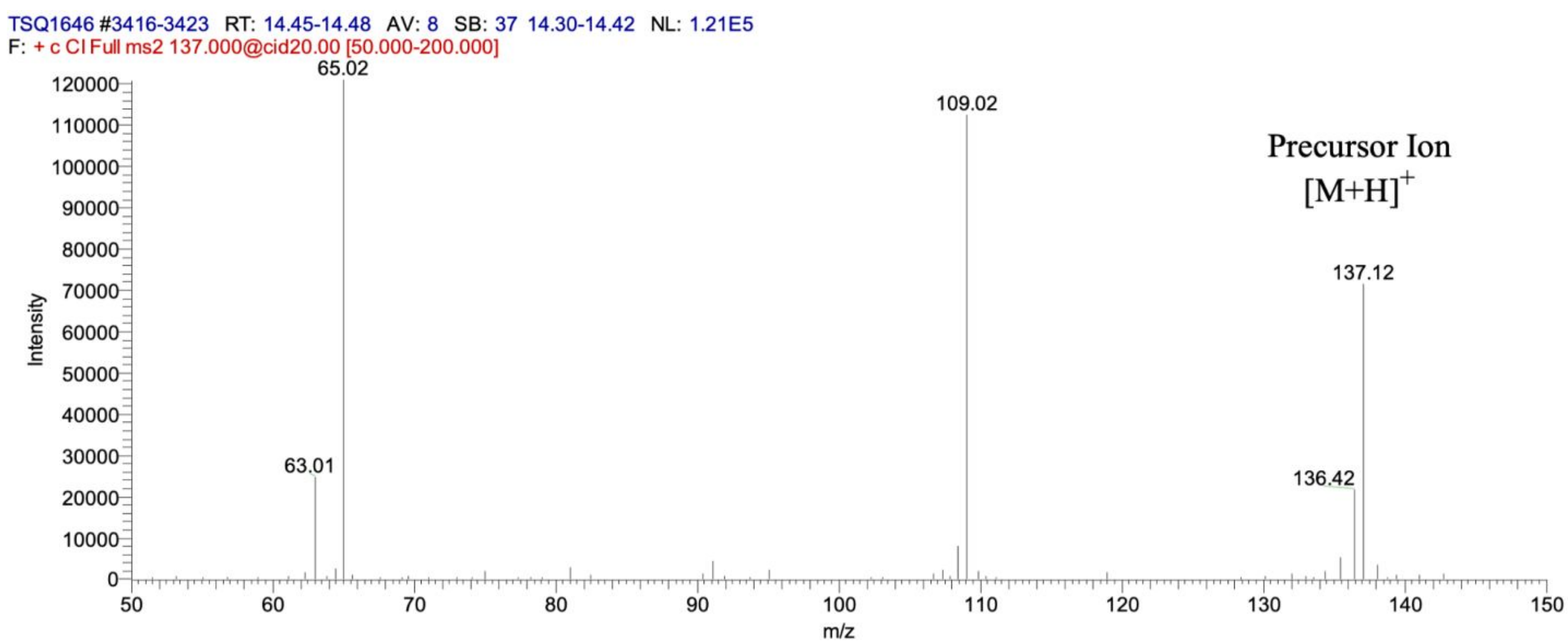

Figure S4. GC-MS-MS (CI) mass spectrum of 1,4-oxathiane 4,4-dioxide standard showing a match to the unknown's mass spectrum in Figure S2.

LC-MS-MS Analysis

LC-MS-MS was conducted in a Thermo Scientific Vanquish Flex HPLC with a Thermo Scientific Q Exactive HF-X. The LC column used for the analysis was a Waters Acquity HSS T3, 1.8 µm. Ultra-high purity nitrogen served as the collision gas. Mobile phases used were A = water/0.1% formic acid and B = acetonitrile/0.1% formic acid. The solvent gradient was 25 minutes long as it was as follows: initial 1%, hold for 2 minutes then ramp to 10% B over 6 minutes; ramp to 95% B over 7 minutes, hold at 95% B for 2 minutes and re-equilibrate at 1% B for 8 minutes. Samples (volume size = 10 µL) were injected into the LC-MS for analysis. The oven temperature was set at 40°C and the MS was operated to scan from m/z = 75 to 750 with a solvent delay of 3.5 min. MS acquisition was performed on a Thermo Scientific Q Exactive HF-X mass spectrometer operated using heated electrospray ionization (HESI) in positive ion mode. The MS experiment is composed of a full MS spectrum (m/z = 75-750) at a resolution of 30,000 at m/z = 200 followed by Data Dependent MS/MS with normalized higher-energy collisional dissociation energies of 30% at a resolution of 30,000 at m/z = 200.

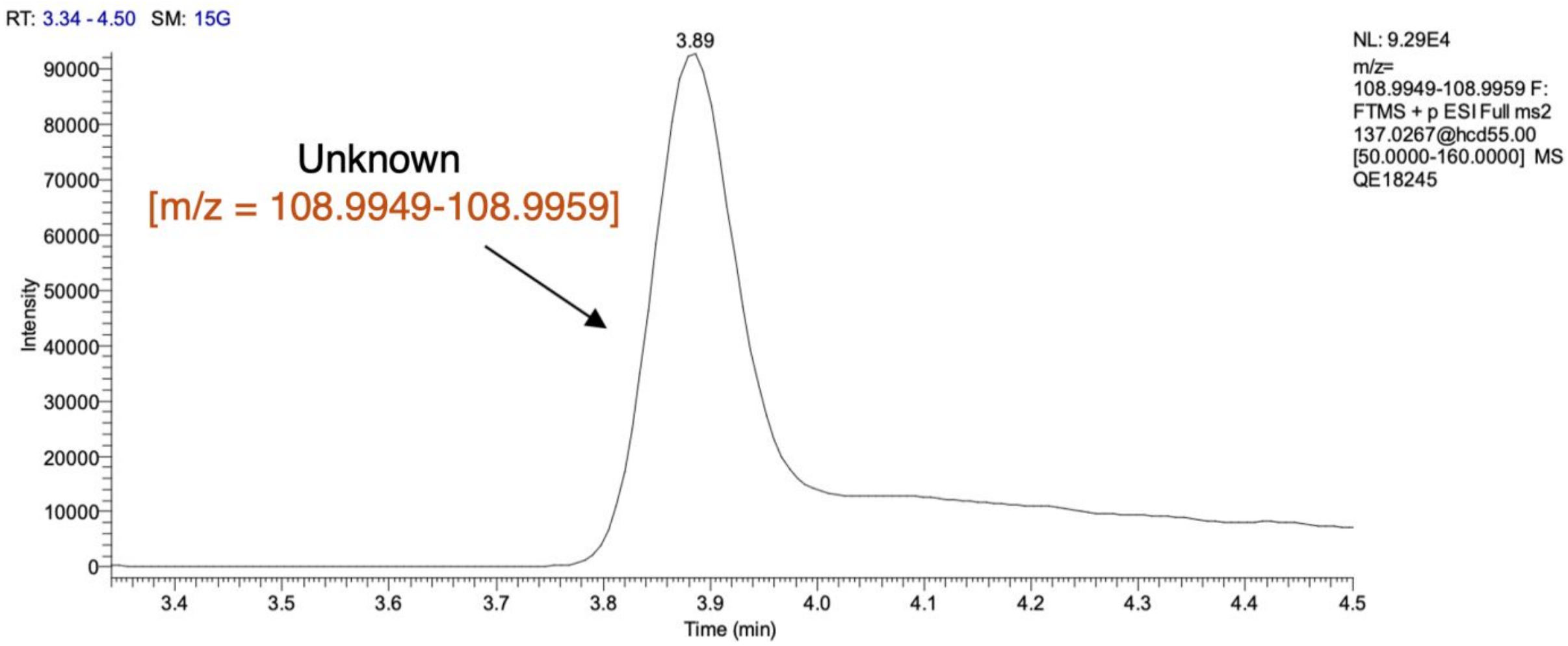

Figure S5. LC-MS-MS selected ion chromatogram of the unknown compound in the S1 matrix.

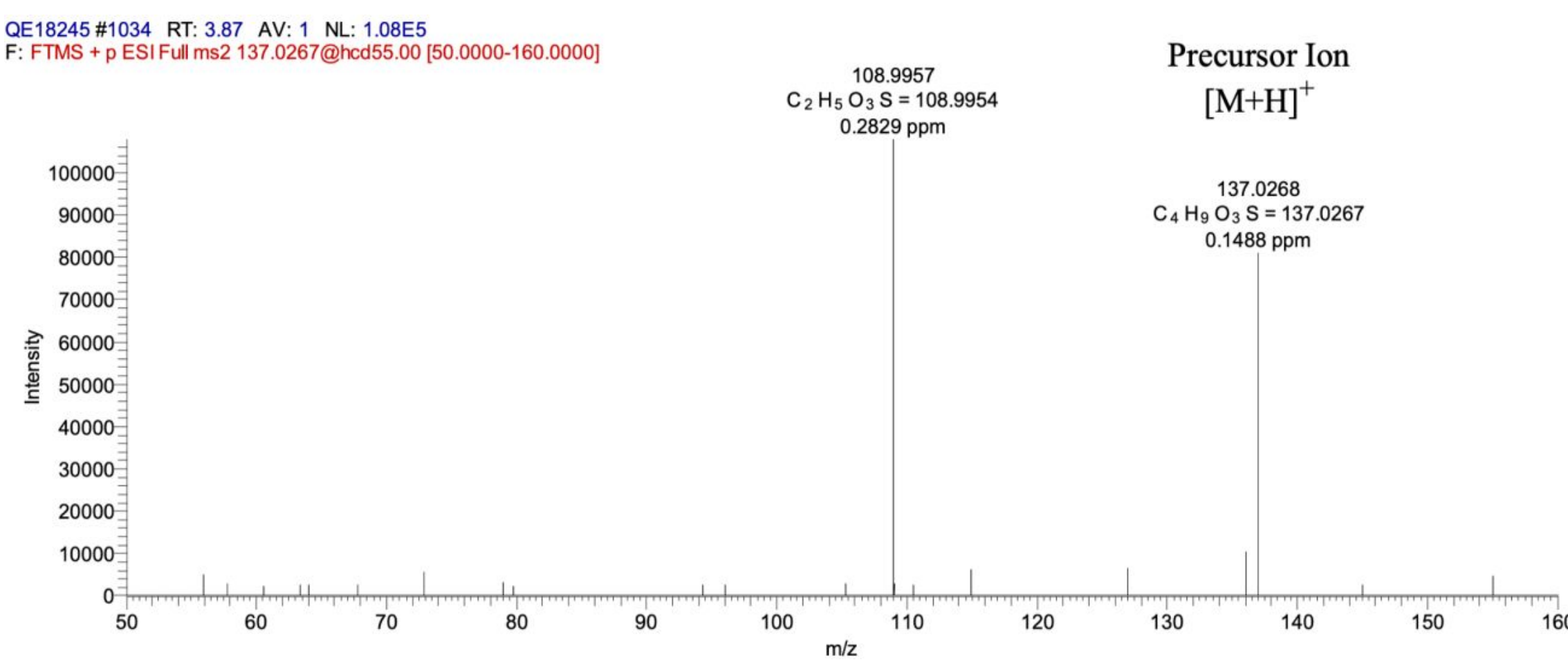

Figure S6. LC-MS-MS mass spectrum for unknown compound in the S1 matrix. The $[M+H]^+$ ion (137.0268) provides the molecular formula $C_4H_8SO_3$ (for the non-ionizied chemical).

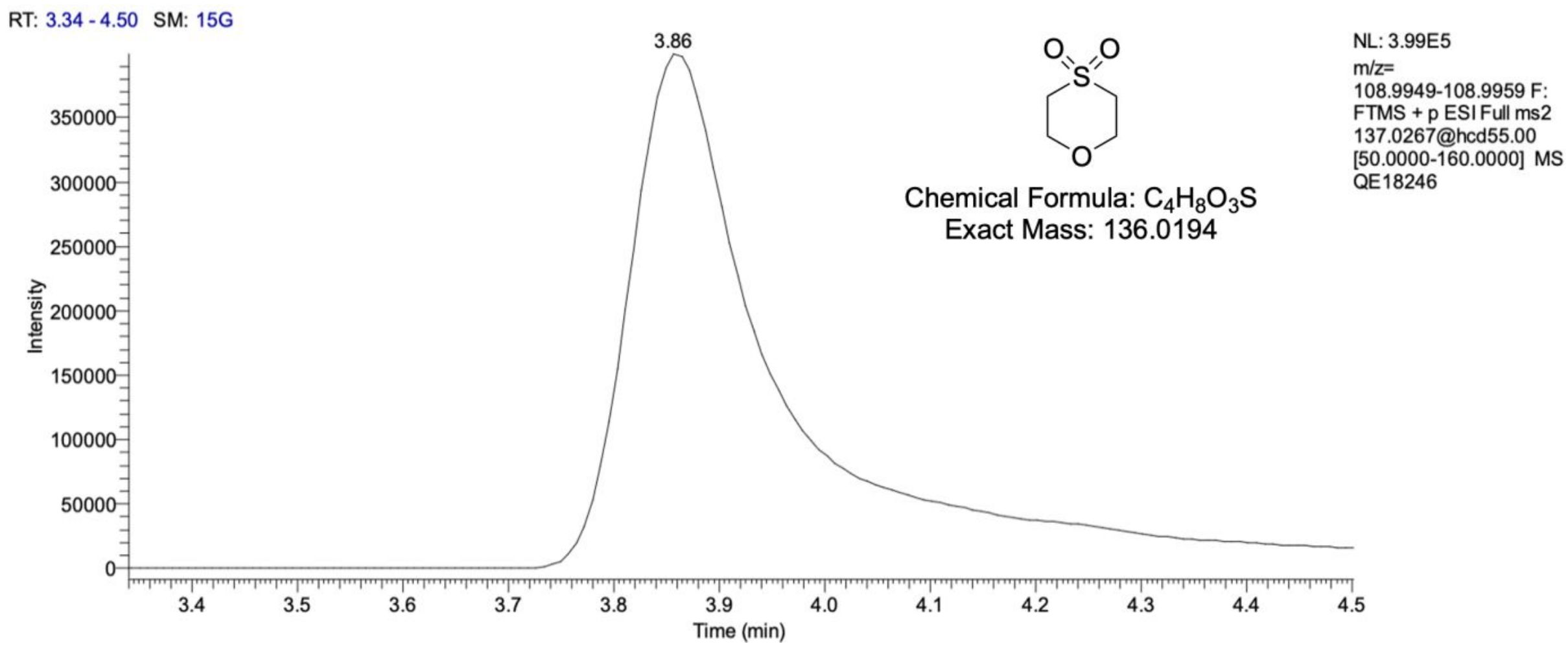

Figure S7. LC-MS-MS selected ion chromatogram of the 1,4-oxathiane 4,4-dioxide standard.

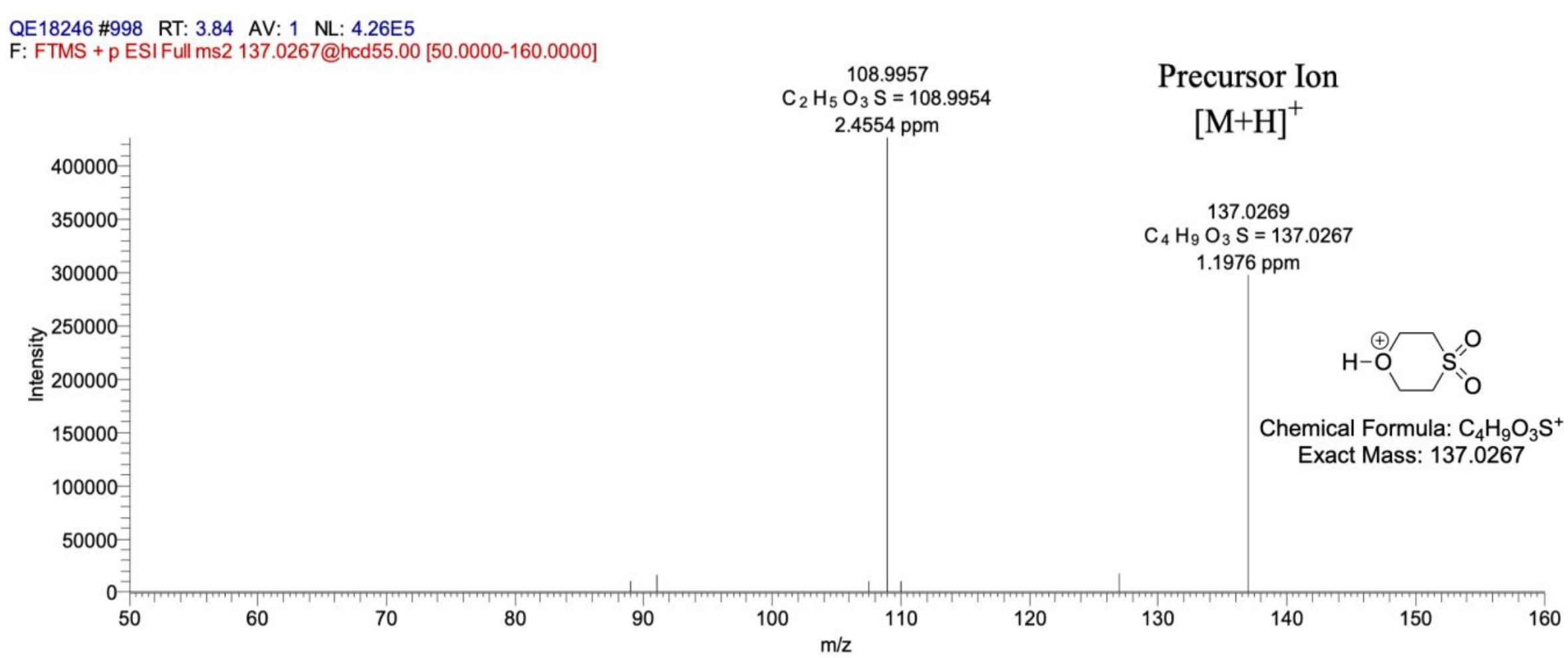

Figure S8. LC-MS-MS mass spectrum for 1,4-oxathiane 4,4-dioxide standard. The calculated exact mass is $[M+H]^+ = 137.0267$, leading to a molecular formula of $C_4H_8SO_3$ for the non-ionized chemical.

Synthesis of 2-hydroxyethyl vinyl sulfone

The synthesis of 2-hydroxyethyl vinyl sulfone was carried out by exposing 2-chloroethyl vinyl sulfone to various basic conditions in acetonitrile in the absence and presence of water as outlined in Scheme S1 below. It was found that the best yield of 2-hydroxyethyl vinyl sulfone was obtained by treating the 2-chloroethyl vinyl sulfone with 0.1 N NaOH (1 equiv.) in acetonitrile at room temperature for 2 hours (41% isolated yield). Other conditions tested are given in Table S1.

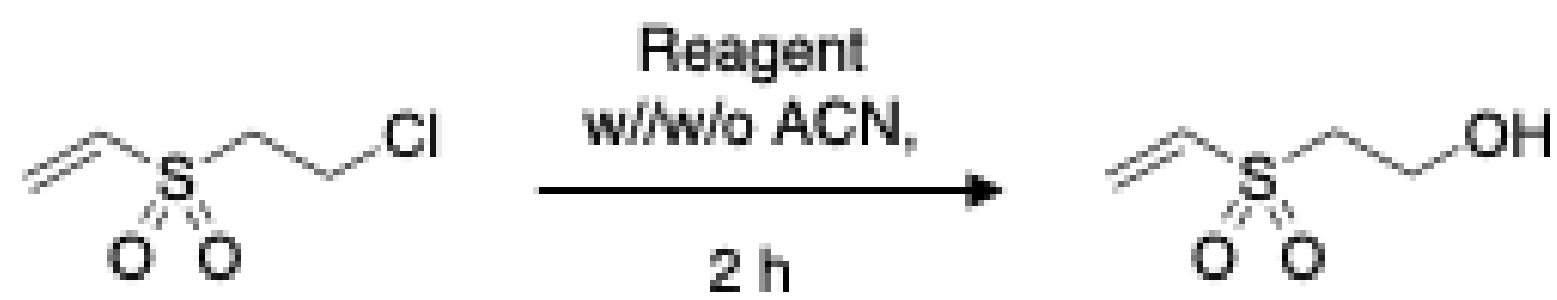

Scheme S1. Synthesis of 2-hydroxyethyl vinyl sulfone

Table S1. Explored conditions for the synthesis of 2-hydroxyethyl vinyl sulfone. Yields refer to isolated ones after flash column chromatography purification.

Reagent (100 μL)	ACN (μL)	Temp (°C)	Yield (%)
0.1 N NaOH	500	24	41
$NaHCO_3$	500	24	22
$NaHCO_3$	500	60	--
$NaHCO_3$	--	60	--
H_2O	--	60	--

Synthesis of 1,4-oxathiane 4,4-dioxide

The synthesis of 1,4-oxathiane 4,4-dioxide was accomplished by oxidizing 1,4-oxathiane under various conditions. Two oxidants were used to carry out the reaction, *m*-chloroperbenzoic acid (mCPBA) and 30% hydrogen peroxide solution. In addition, different solvents were evaluated such as DCM, $CHCl_3$, MeOH and NMP. The best yields for 1,4-oxathiane 4,4-dioxide were obtained when using mCPBA at 65 °C in DCM or $CHCl_3$ for 2 hours (33-38% yield).

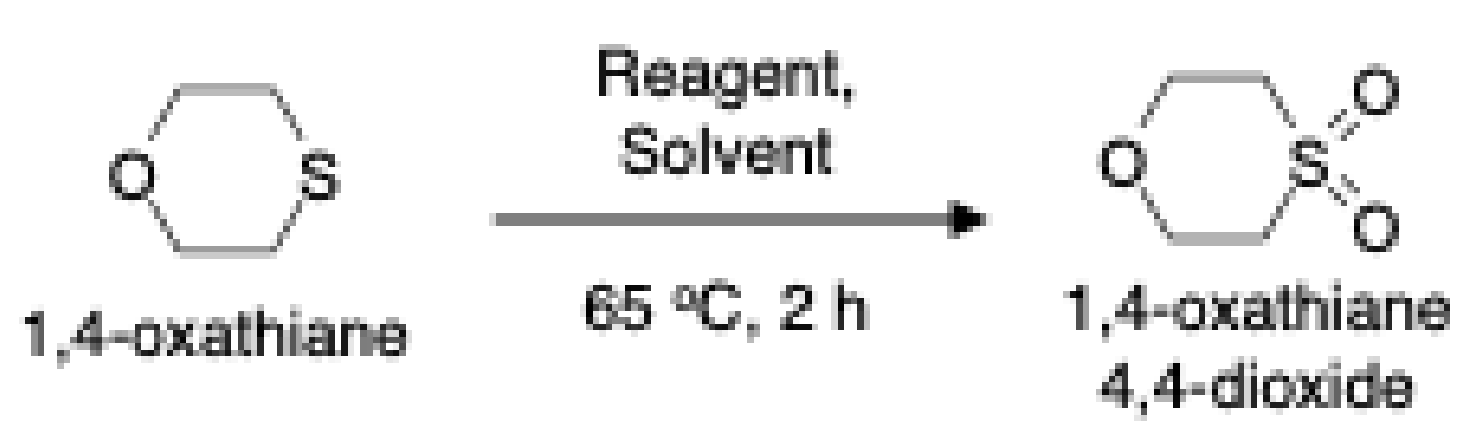

Scheme S2. Synthesis of 1,4-oxathiane 4,4-dioxide

Table S2. Oxidation conditions for 1,4-oxathiane for the synthesis of 1,4-oxathiane 4,4-dioxide.

Reagent	Solvent	Yield (%)
m-CPBA	DCM	33
m-CPBA	$CHCl_3$	38
30% H_2O_2	--	11
30% H_2O_2	MeOH	18
30% H_2O_2	NMP	14

Additional differences in expanded sections of the mass spectra for both, 1,4-oxathiane 4,4-dioxide and 2-hydroxyethyl vinyl sulfone can be observed below.

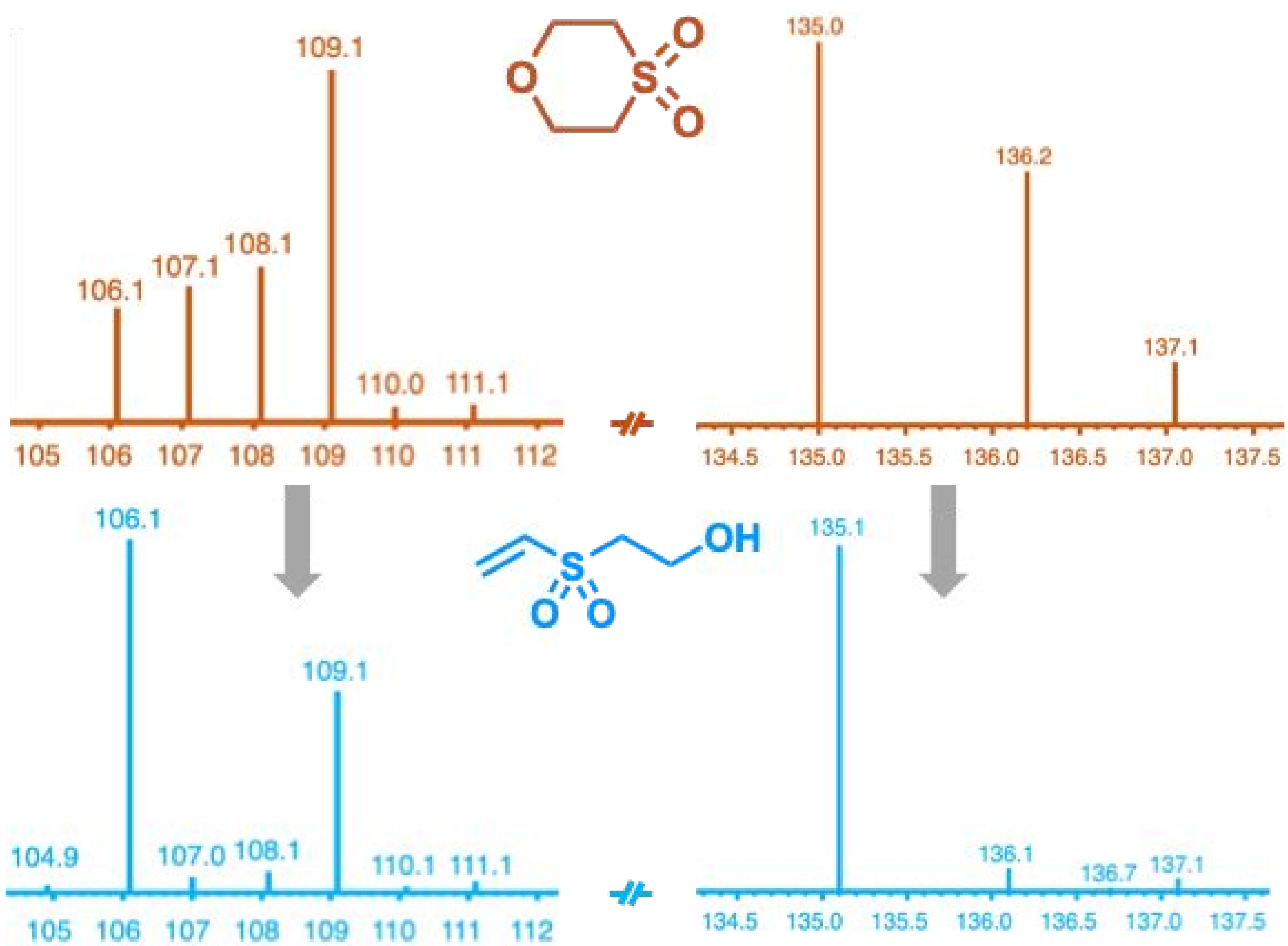

Nuclear Magnetic Resonance

Spectra were obtained using a Bruker Avance III 600 MHz instrument equipped with a Bruker QNP 5 mm cryoprobe (Bruker Biospin, Billerica, MA.) at 30.0 ± 0.1°C. ^{1}H NMR (600 MHz) was carried out in $CDCl_3$. Chemical shifts are calibrated with respect to the residual $CHCl_3$ peak centered at 7.26 ppm.

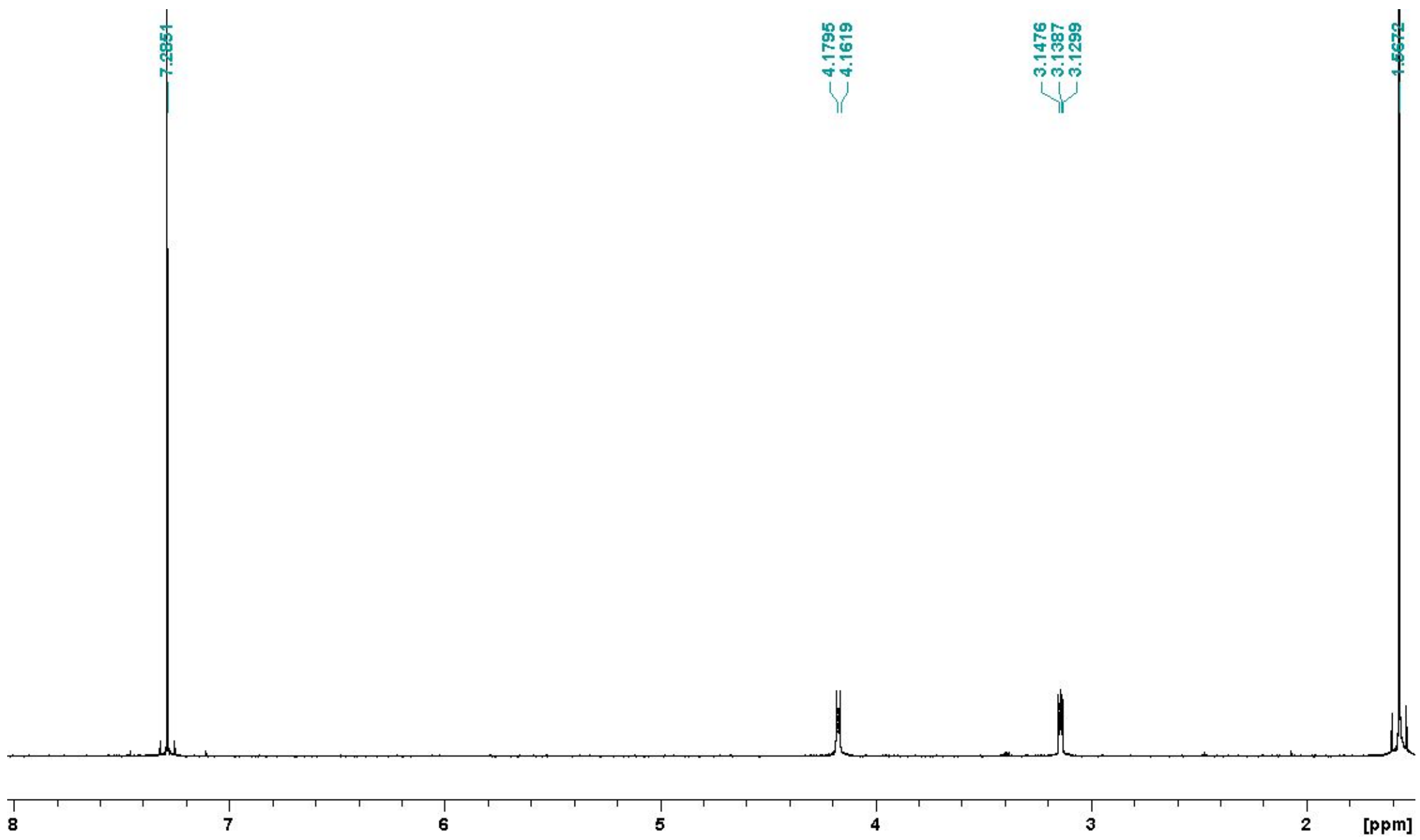

Figure S9. ^{1}H NMR ($CDCl_3$) spectrum of 1,4-oxathiane 4,4-dioxide.

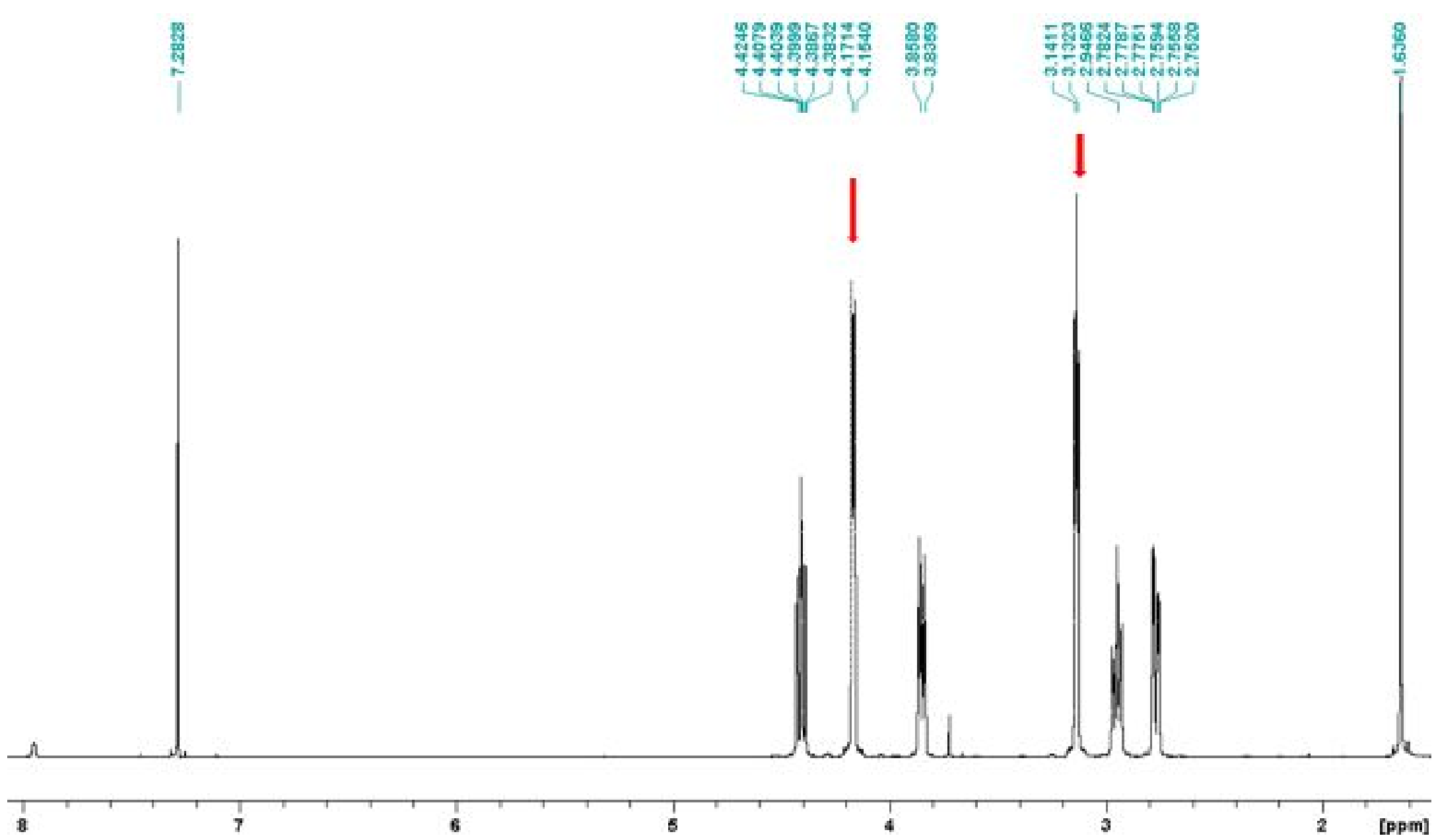

Figure S10. ^{1}H NMR (with $CDCl_3$ solvent) spectrum of sample S1 with the peaks corresponding to 1,4-oxathiane 4,4-dioxide denoted by red arrows.
